# Geometric conductive filament confinement by nanotips for resistive switching of HfO_2_-RRAM devices with high performance

**DOI:** 10.1038/srep25757

**Published:** 2016-05-16

**Authors:** Gang Niu, Pauline Calka, Matthias Auf der Maur, Francesco Santoni, Subhajit Guha, Mirko Fraschke, Philippe Hamoumou, Brice Gautier, Eduardo Perez, Christian Walczyk, Christian Wenger, Aldo Di Carlo, Lambert Alff, Thomas Schroeder

**Affiliations:** 1 IHP GmbH – Leibniz institute for innovative microelectronics, Im Technologiepark 25, 15236 Frankfurt (Oder), Germany; 2 Electronic Materials Research Laboratory, Key Laboratory of the Ministry of Education & International Center for Dielectric Research, Xi’an Jiaotong University, Xi’an 710049, China; 3 Dept. Electronics Engineering University of Rome, “Tor Vergata” via del Politecnico 1, 00133 Roma, Italy; 4 Institut des Nanotechnologies de Lyon (INL), UMR CNRS 5270, INSA de Lyon, 7 Avenue Jean Capelle, 69621 Villeurbanne, France; 5 Institute of Materials Science, Technische Universität Darmstadt, 64287 Darmstadt, Germany; 6 Brandenburgische Technische Universität, Konrad-Zuse Strasse 1, 03046 Cottbus, Germany

## Abstract

Filament-type HfO_2_-based RRAM has been considered as one of the most promising candidates for future non-volatile memories. Further improvement of the stability, particularly at the “OFF” state, of such devices is mainly hindered by resistance variation induced by the uncontrolled oxygen vacancies distribution and filament growth in HfO_2_ films. We report highly stable endurance of TiN/Ti/HfO_2_/Si-tip RRAM devices using a CMOS compatible nanotip method. Simulations indicate that the nanotip bottom electrode provides a local confinement for the electrical field and ionic current density; thus a nano-confinement for the oxygen vacancy distribution and nano-filament location is created by this approach. Conductive atomic force microscopy measurements confirm that the filaments form only on the nanotip region. Resistance switching by using pulses shows highly stable endurance for both ON and OFF modes, thanks to the geometric confinement of the conductive path and filament only above the nanotip. This nano-engineering approach opens a new pathway to realize forming-free RRAM devices with improved stability and reliability.

Low energy dissipation makes resistance switching random access memory (RRAM) a very interesting non-volatile storage media for wireless sensor networks^1^ and neuromorphic applications^2^. For such applications, where the integration intensity issue is not as severe as for “beyond Flash” technologies, the production of RRAM modules is mainly delayed by resistance switching reproducibility (cycle-to-cycle and device-to-device)^3^. Resistance switching has been investigated in many binary oxides, the most important ones from a technology point of view are NiO^4^, ZnO^5^, TiO_2_^6^, Ta_2_O_5_^7^ and HfO_2_^8^ using metal/oxides/metal structures. Among them, hafnium oxide is of special technological interest due to the availability of deposition tools and processes for Si CMOS front-end applications like high-k gate dielectrics for advanced CMOS technologies.

Over the last years, significant progress has been made in understanding the resistance switching mechanism in HfO_2_. It is widely accepted that low/high resistance states are achieved by forming/breaking a few nanometer wide conductive filament (CF), bridging/non-bridging the electrodes as a function of an applied external electrical field^3^. Nanoionic redox processes at the oxide/electrode switching interface and ion transport allow modulating the resistance of the oxide between two or more states. The ON- and OFF-states correspond to the low resistance state (LRS) and the high resistance state (HRS), respectively. Physicochemical analyses^9^,^10^, electrical measurements^11^,^12^,^13^ and *ab-initio* simulations^14^ performed on HfO_2_ have shown that the CF responsible for the resistance switching consist of a chain of oxygen vacancies (V_O_). Particularly, the variation of the HRS state is a general problem because uncontrolled V_O_ distribution in RRAM cells leads to cycle-to-cycle variation of HfO_2_ insulating states and filament growth^15^. Based on the current physics understanding, a better control of V_O_ distribution dynamics is necessary in order to improve resistance state variations for the reproducible cycle-to-cycle device operation^16^.

In order to control the V_O_ generation and distribution, so that the CF is confined along the same path at each voltage stress^15^,^16^, several approaches have been suggested such as doping^17^,^18^, introducing nanoparticles^19^,^20^, using bilayers of different oxides^21^ and employing “nanovia” electrode structures^22^. In case of HfO_2_-based doping approach, the chemical stabilisation of the filament is believed to occur along the trivalent dopant atoms (e.g. Al^3+^)^17^,^18^. Ru and Pt nanoparticles have been embedded in TiO_2_ sandwiched between two Pt electrodes^19^,^20^ to realize the local field enhancement so that the filament forms at the particle location. In the bilayer approach, two switching oxides, for instance ZrO_2_ and HfO_2_^21^, are deposited one on top of each other so that the resistance switching region is limited to the ZrO_2_/HfO_2_ interface. Above-mentioned three methods could all provide narrow ON- and OFF-states distribution. However, controlling the morphology and the spatial distribution of the dopants and the nanoparticles is very challenging, which again might lead to process complexity and to important device-to-device variation over the wafer. Therefore, nanodevices like Pt/HfO_2_/TiN-nanovia structures with a better control of the nm-size electrodes have been also reported to offer electrode geometry related filament confinement^22^. Nevertheless, the resistance states dispersion was not discussed in detail and all above-mentioned approaches are not yet compatible with the current complementary metal–oxide–semiconductor (CMOS) technology. In this work, we present an effective geometric approach by nanotip electrodes processed by CMOS compatible technology to confine the filament location by localized electrical field enhancement effects so that we are able to report highly stable endurance and retention of HfO_2_ RRAM devices.

## Results and Discussion

**Figure 1:**
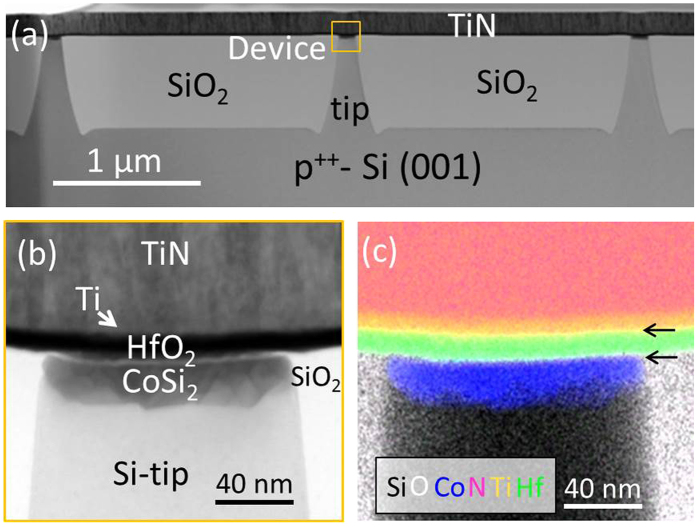
STEM-EDX performed on the cross-section of a TiN/Ti/HfO_2_/CoSi_2_/Si tip device. (**a**) STEM image overview showing three Si tips; (**b**) High magnification STEM image on the squared device part in (**a**). (**c**) EDX chemical analysis of the same region in (**b**) and the element of Si, O, Co, N, Ti and Hf are represented by the color of black, white, blue, pink, orange and green, respectively. The arrows in (**c**) mark the partly oxidized Ti and CoSi_2_ layer.

We study a TiN/Ti/HfO_2_/CoSi_2_/Si-tip device, where the CoSi_2_ bottom electrode is locally deposited at the top of the Si nanotip. Structural and chemical analyses were performed by employing transmission electron microscopy (TEM) and energy-dispersive x-ray spectroscopy (EDX). The results are displayed in Fig. 1. Figure 1a shows an overview of the device, which demonstrates that the Si tips locate in a SiO_2_ layer and on top HfO_2_ and Ti/TiN layers were deposited. The device part is marked by a square. In order to observe more details, an image was performed at higher magnification for the squared part in Fig. 1a and is shown in Fig. 1b. It indicates clearly that the Ti/HfO_2_ and the HfO_2_/CoSi_2_ interfaces are abrupt, which lead therefore to a good electrical contact between electrodes and the HfO_2_ layer. Moreover, it can be noticed that the surface of the Si tip (with a diameter of ~120 nm) is very flat on nm scale. In Fig. 1c, elemental mapping has been performed. The layers forming the device are clearly identified, no inter-diffusion is observed within the sensitivity range of the EDX analysis. It can also be observed that thin (several nm) oxygen rich layers exist at the Ti/HfO_2_ and HfO_2_/CoSi_2_ interface (the white layers marked by arrows in Fig. 1c), which formed during film deposition and indicate that both Ti and CoSi_2_ could serve as oxygen reservoir layers during the RS of the device^10^,^23^.

Furthermore, it has to be noted here that different from 1-transistor-1-resistor (1T1R) RRAM devices where a post-deposition annealing (PDA) at a temperature of ~400 °C is generally carried out in order to improve electrical contacts and conductivity of metal lines^8^,^24^, no PDA process was performed in nanotip devices presented in this work. Firstly, PDA at ~400 °C will result in the recrystallization of HfO_2_ thus forming many grain boundaries in the film, which could influence the CF formation^25^,^26^. This is clearly not our case. As shown Fig. 1b, HfO_2_ grown at 320 °C without PDA remains amorphous without detectable crystallites^9^. Secondly, PDA annealing will also lead to a much more severe Ti oxidation by oxygen scavenging form the HfO_2_ layer before the electrical measurements, so that typically the oxygen reservoir is positioned on the top Ti/HfO_2_ interface and clockwise switching behaviour (set at positive, reset at negative voltage) is in consequence observed^27^. As will be discussed in the following, counter clockwise switching behaviour is observed in the here discussed system.

**Figure 2:**
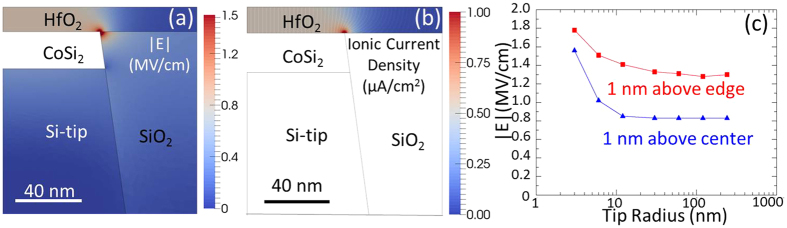
FEM calculated maps of Ti/HfO_2_/CoSi_2_/Si-tip heterostructure on a 2D slice in 3D cylinder coordinates of (**a**) the electrical field distribution and (**b**) the ionic current density (V_O_) distribution. (**c**) Evolution of the electric field |E| in the ~10 nm HfO_2_ film at the sites 1 nm above the center (blue triangles) and above the edge (red squares) as a function of the tip radius.

To evaluate the geometric confinement of the V_O_ distribution by the nanotip electrode, numerical calculations using finite element method (FEM) were carried out and the results are shown in Fig. 2. More simulation details can be found in the experimental part. A simplified geometry assuming a perfect cylindrical symmetry is adopted and we calculated on a 2D slice in cylinder coordinates. The layers thicknesses and the tip diameter measured on the STEM image displayed in Fig. 1 (t(HfO_2_) = 9 nm; t(CoSi_2_) = 20 nm; tip radius ~ 60 nm) were used to design the model. Figure 2 (a) shows a typical electric field distribution map obtained by solving the Poisson solution. The electrostatic potential difference between Ti/CoSi_2_ was set to 0.7 V. The permittivity values of ɛ(HfO_2_) = 16 and ɛ(SiO_2_) = 3.9 were used. Both the top metal and the CoSi_2_ has been modeled as ideal metals with infinite conductivity and therefore substituted by Dirichlet boundary conditions for the electric potential. The TiN/Ti top electrode is not shown in the model shown in Fig. 2 but it is included as a boundary condition setting the fermi level for Ti metal. It can be evidently observed in Fig. 2(a) that the electrical field is well confined on top of the nanotip area (pink region, |E| = 0.8 MV/cm). Moreover, the electrical field demonstrates an obvious enhancement at the edge part of the nanotip (red region, |E| = 1.4 MV/cm). This E enhancement will probably lead to the favorable location for V_O_ filament formation at the nanotip edges. To clarify this point, we investigate the ionic current density (j) distribution map using the E field obtained from Fig. 2(a). At the initial stage of the filament formation, we assume that there is a spatially homogeneous density of V_O_ of 10^19 ^cm^−3^ with a diffusion constant of ~10^−14 ^cm^2^/s (see ref. 28) and the ionic current density is purely drift driven by the electrostatic field, according to j = q^2^/(k*T) *D*n*E, where q is the elementary charge, k Boltzmann constant, T the temperature, n the V_O_ density and E the electric field. The ionic current density map and flow lines are shown in Fig. 2(b), which indicates that the current pathway is also confined on nanotip surface region (j = 0.5 μA/cm^2^) and the edge region serves as a preferential starting point of the current flow with an enhancement of ionic current density of j = 0.9 μA/cm^2^.

Let us now consider the difference of the nanotip electrode method compared to the conventional planar “sandwich-like” MIM structure method and its possible effects on the RS. Firstly, because the planar “sandwich-like” device has a symmetric top and bottom electrodes configuration, during the forming process, the V_O_ forms in the ideal case randomly and homogeneously in the whole HfO_2_ film. Once the applied voltage reaches a critical value (forming voltage), the V_O_ density reaches the percolation value so that a filament consisting of a chain of V_O_ is established in the HfO_2_ film (soft breakdown)^29^. Again, due to the symmetric planar device structure, the filament might locate randomly at arbitrary site of the electrodes and for different cycles it can be located along different paths in the oxide film. It is well-known that the filament size and shape influence the resistance states so that strong variations in these parameters might result in LRS as well as HRS instabilities. For example, it was shown by Chen *et al*.^30^ that a localized narrow constriction in the filament is beneficial for stable switching conditions (high endurance) whereas a broad constriction is of advantage for stable retention (limiting the effect of uncontrolled V_O_ out diffusion). In contrary, in the nanotip electrode device structure discussed in this work, as we already showed (Fig. 2), the V_O_ generation and distribution are geometrically well confined on the nanotip surface. Moreover, the edge of the nanotip (in fact a “ring” region) offers a preferential generation region for the filament with a narrow constriction for stable endurance due to the enhancement of the electric field and the ionic current density in this part. That is to say, the V_O_ can only distribute in the HfO_2_ film part which is on the nanotip area (in this case a cylinder volume with bottom circle radius of 60 nm) and the filament initially generated at the edge of the nanotip can exist only in this volume. Depending on the different experimental condition, the filament can have different sizes (with diameter ranging from several nm to several hundred nm)^9^,^31^,^32^,^33^,^34^ but always locates on nanotips.

The impact of the nanotip radius on the geometric filament confinement is of great interest and thus we investigated the field enhancement as a function of tip radius, ranging from 2 nm to 300 nm. The detailed E maps can be found in the Supplementary Information. Figure 2(c) shows the fields in the HfO_2_ film measured above the center (blue triangles) and above the edge (red squares) of the nanotip, 1 nm away from the HfO_2_/nanotip interface. It can be seen that when the tip radius is larger than the HfO_2_ film thickness of ~10 nm, the |E| values above the center and above the edge both show constant values of 0.8 MV/cm and 1.4 MV/cm, respectively and the edge shows a clear enhancement of electrical field. When the tip radius is smaller than the HfO_2_ film thickness of ~10 nm, both |E| values above the center and above the edge increase and the difference between them become much less pronounced, which leads to a classical nanotip enhancement. It is worth pointing out that, the tip radius of 60 nm and even smaller ones, are comparable with the reported filament sizes^9^,^31^,^32^,^33^ therefore the majority or even the whole nanotip surface would be the effective switching area, with the strongest part certainly located at the edge. We note here that, using reactive ion etching (RIE) processing the nanotip radius can be as small as 2–5 nm and the final tip surface size can be well tuned by chemical mechanical polishing (CMP) process time, which would allow tuning the operation voltage of forming, set and reset by nanotip engineering depending on the application. In addition, the fabrication of Si nanotips does not require expensive lithography tools. From the devices scaling point of view, the nanotip method presents a good way to study nm scale RRAM devices and demonstrates competitive advantages (tunable and production cost).

**Figure 3:**
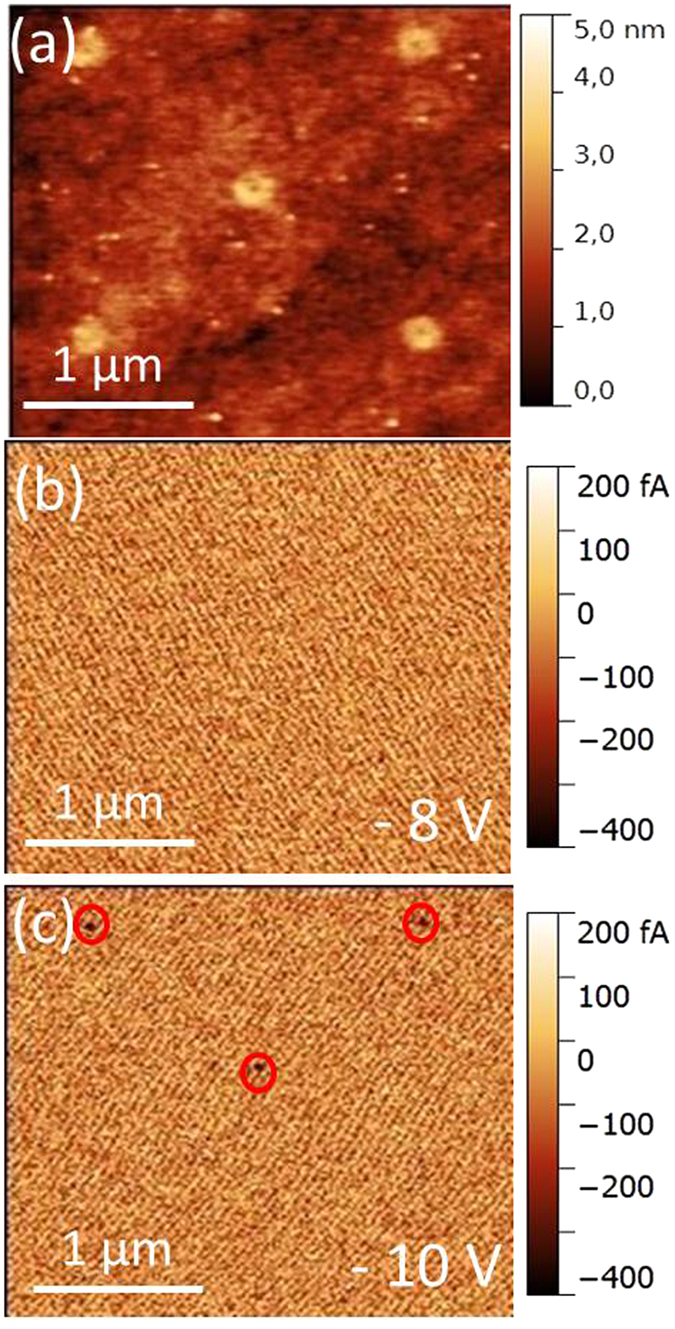
AFM measurements performed on the HfO_2_ surface without top electrodes. (**a**) Topography. (**b**,**c**) Current maps (TUNA) with bias of −8 V and −10 V, respectively. The black spots marked by red circles in (**c**) are conductive spots (the end of CFs) on the nanotip locations.

In order to confirm the filament confinement above the tips experimentally, tunnelling atomic force microscopy (TUNA) has been performed on a device processed up to the HfO_2_ layer (no top electrode). Figure 3a displays a topography image of HfO_2_ surface, which allows locating the tips underneath the surface by the presence of donut-shaped protuberances around the supposed location of the tip (diameter 250 nm). This effect is due to the CMP process, which is more effective for SiO_2_ than for Si. Figure 3b,c show the current maps performed by polarizing CoSi_2_ (AFM tip was grounded) with two different voltages −8 V and −10 V, respectively. In Fig. 3b, for the current map with lower bias (−8 V) no conduction spot was measured, even in the thinnest parts of the oxide film, indicating the good insulating properties of the HfO_2_ film. In Fig. 3c, by using a higher bias (−10 V), conduction spots (with I_leakage_ ~ −400 fA) appear at the several tip locations (marked by red circles). This means the CFs formed at these locations in the film. The confined leakage currents suggest that, in the full processed device, the filament would preferably form above the tips. It can be noticed that conduction spots do not appear on every tip location, due to small process variations leading to the material inhomogeneity of the oxide film and/or nanotip surfaces. A further increase of the forming voltage would induce the CF formation on each tip^35^. We note here that due to the small size of the nanotip radius (~60 nm, comparable with the filament size) and TUNA resolution limit using an AFM tip with radius of ~30 nm, it is impossible to discriminate between location of the conducting filament either on the tip surface or at the tip edge. More importantly, it should also be noted that the current maps shown here were obtained by individually scanning the Si nanotip electrodes under the HfO_2_ film by the AFM tip with certain bias, which is different from the electrical measurements by simultaneously applying electrical field on the TiN/Ti top electrode covering many tips. However, the above-shown inhomogeneous appearance of the conduction spots indicates that, for the electrical measurements with some certain bias on TiN/Ti/HfO_2_/CoSi_2_/Si-tips including a large number of tips, the filament will be preferentially formed on one or several tip regions showing the “weak points”. That is to say, not all tips will be active in the switching process and the nanotip approach can thus be viewed as a way to achieve stable switching condition by parallelization in the present case.

**Figure 4:**
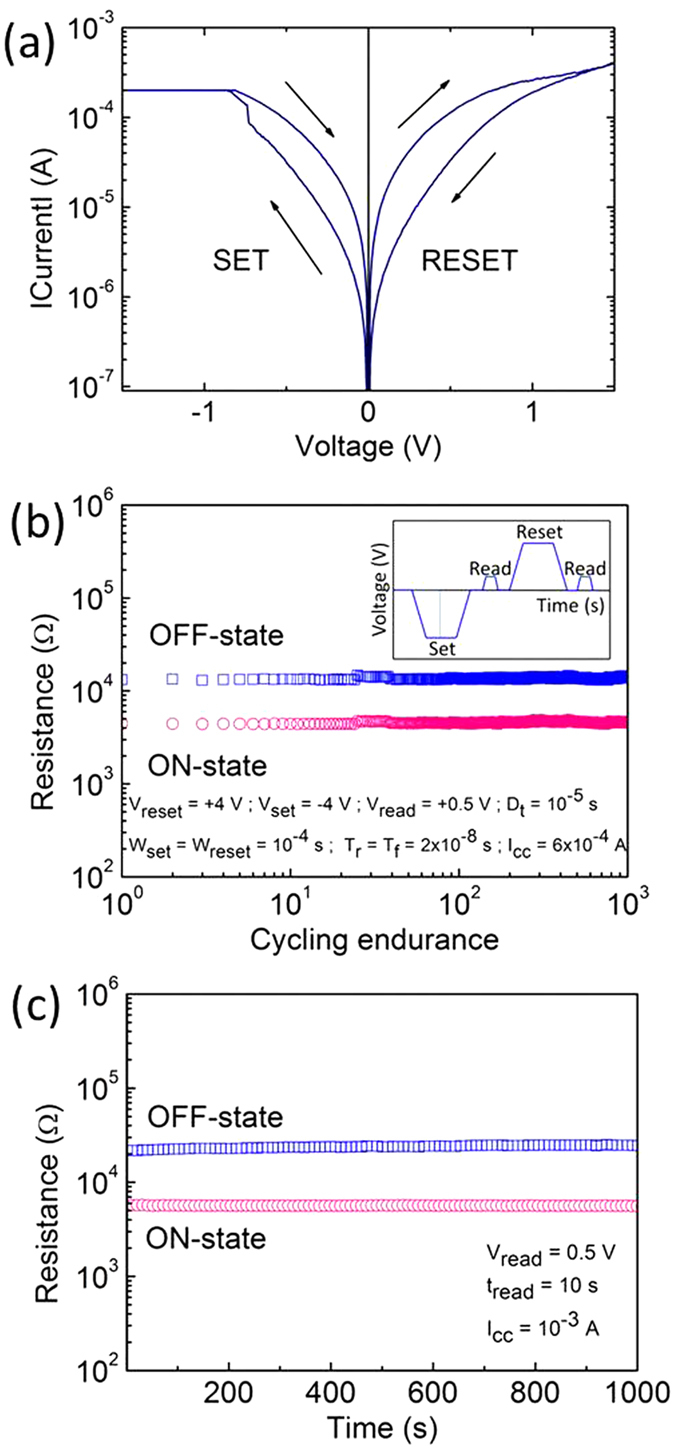
Electrical characterization of the TiN/Ti/HfO_2_/CoSi_2_/Si-tips device. (**a**) Typical DC sweeps showing the evolution of the current intensity as a function of the voltage. (**b**) Pulse cycling endurance showing the evolution of the OFF- and ON-resistance states as a function of the number of cycles. The experimental details are shown in the inset and remarks in the figure. (**c**) Retention measurement performed at room temperature with experimental details shown in the figure remarks.

In order to confirm the resistance switching and a good control over the resistance state dispersion along cycling, the TiN/Ti/HfO_2_/CoSi_2_/Si-tips device has been characterized electrically and the results are shown in Fig. 4. Figure 4a shows typical I-V characteristics (DC sweep mode) performed by applying the voltage on the top electrode. It should be firstly noted here that the additional forming process done by the first set process, which is normally required in RRAM devices with planar electrodes, is not needed for our nanotip devices. This behaviour is certainly related to the good control of the V_O_ generation and distribution and the electrical field enhancement on the tip edges (almost double) by using our method. In the planar electrode devices, the whole Ti top metal layer is oxidized during forming in order to generate enough V_O_ with density higher than the percolation value, thus higher energy is needed in the first device forming cycle than in the following set/reset cycles. In nanotip method, it is probable that only the limited Ti and CoSi materials being vertically above the tip area is oxidized during the initial switching cycle to generate the filament.

In Fig. 4a, in the negative polarity, below −1 V, the current absolute value increases quickly until it reaches the compliance (2 × 10^−4^ A). The polarity is an issue of interest in RRAM devices. As a matter of fact, even for the devices with same layer stacking, e.g. TiN/Ti/HfO_2_/TiN, different polarities have been reported in 1T1R integrated devices^8^,^24^,^30^,^36^ and single devices^10^,^37^,^38^,^39^. Single devices show a negative polarity of the set process whereas in 1T1R devices where a PDA was performed after film deposition which leads to more severe pre-oxidation of Ti thus a positive set process. Here in our nanotip devices, no additional PDA was performed thus they demonstrate a negative polarity of set, namely, the rupture of the filament during reset occurs at the Ti/HfO_2_ interface and the root of filament with a conical shape^30^,^34^,^40^,^41^,^42^ is at the HfO_2_/CoSi_2_ interface. A compliance current (I_cc_) of 2 × 10^−4^ A was chosen because lower I_cc_ leads to instable RS. It is widely reported^7^,^17^,^38^,^43^,^44^,^45^ that I_cc_ can strongly influence the RS and too low I_cc_ possibly results in instable filaments (due to thermally activated V_O_ out-diffusion effects). In our case, it is possible that lower I_cc_ generates filaments much thinner than the tip surface diameter (probably on the nanotip edge region due to the ionic current density enhancement there) thus not stable and the filament size with I_cc_ of 2 × 10^−4^ A is larger (covering more area of the tip surface) thus more stable^34^,^46^. On the back sweep, the low resistance state is maintained, confirming the writing of the information (set). Using a positive polarity, above +1 V, the device is switched back to the high resistance state (reset). Thus, the device exhibits bipolar resistance switching at low voltage/power values. The resistance ratio (read at +0.5 V) is 3.4 in the shown case which is typical for nano-devices^30^.

In the final high-end applications of RRAM, the pulse mode is used to operate the devices. It is therefore crucial to measure the resistance state variability using pulses. In Fig. 4b, the device has been cycled using the pulse mode. The inset represents a drawing of the wave form used for pulse resistance switching, which shows the evolution of the voltage applied on the memory cell as a function of the time. Contrarily to the DC mode, the voltage is not progressively increased, but the voltage is rather a constant and the polarity changed abruptly for reset and set. The complete wave form is more complex than the drawing in the inset of Fig. 4b. The pulse properties have been carefully optimized in order to get stable memory states. The voltage (V_set_; V_reset_), the delay time between set and reset (D_t_); the pulse width (W_set_; W_reset_), the rise and fall time (T_r_; T_f_) and the I_cc_ are indicated in Fig. 4b. The read voltage (V_read_) was fixed at +0.5 V. 10^3^ pulse cycles have been performed and no significant variation of the resistance was measured. Clearly, the OFF- and ON-states are stabilized at 13.7 kΩ and 4.6 kΩ, respectively. We attribute the highly stable cycle-to-cycle endurance to geometric confinement of filament and V_O_ distribution at the nanotip bottom electrode, as discussed in last parts (Figs 2 and 3). At each set process (with the same I_cc_), the V_O_ aligns along the same path so that no noticeable variation of LRS and HRS occurs. In contrary, I-V characteristics of planar devices containing the same MIM stack of TiN/Ti/HfO_2_/CoSi_2_/Si show much stronger OFF state fluctuations (see more details in the Supplementary Information).

Furthermore, the detailed experimental data shows that the current of ON state is ~110 μA at V = +0.5 V. According to the theoretical estimation using a physics-based analytical Quantum Point Contact (QPC) model, one quantum conductance unit G_0_ (a filament with the diameter of a single atom) is of the order of G_0_ = 2e^2^/h ≈ (12.9 kΩ)^−1^, where e is the electron charge and h is the Planck constant^47^,^48^. This leads to a leakage current of ~39 μA at V = +0.5 V. Therefore, it is probably that 2–3 filaments are simultaneous formed in our working nanotip devices, particularly considering that in our sample the TiN/Ti top electrode covers ~5200 nanotips. This result confirms the observation of TUNA (Fig. 3c) and our related discussion that not all nanotips in the array need to become active in the end in the switching process.

Finally, the device retention has been tested. Figure 4c shows the evolution of the OFF- and ON-states along the time when the device is operated under a voltage stress of +0.5 V at room temperature. The resistance values for both ON- and OFF-states are read every 10 s. Both resistance states are constant, which confirms non-volatile information storage and the formation of a sufficiently broad nano-filament, thus counterbalancing V_O_ out-diffusion effects by thermal processes.

**Figure 5:**
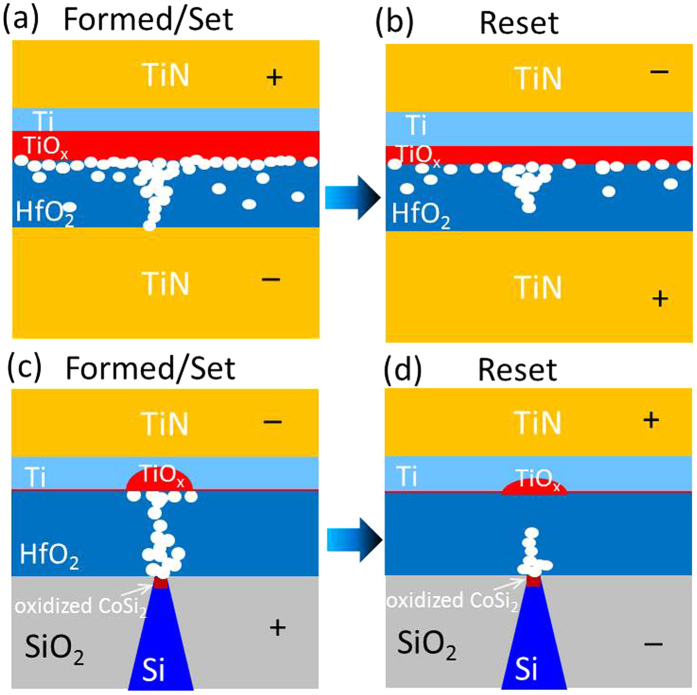
Schematic illustration of the V_O_ (white circles) distribution of planar electrode MIM devices in (**a**) the Formed/Set state and (**b**) the Reset state, in which the V_O_ distribution as well as the filament is not confined thus leading to instable endurance; (**c**) the filament formation of Si-tip electrode MIM devices in the Formed/Set state, in which the V_O_ distribution and the filament is geometrically confined around the tip area; (**d**) the filament rupture during Reset process.

Figure 5 schematically illustrates the material details during RS of both planar and nanotip devices. Figure 5a,b show the status of set and reset states of the planar device, respectively (more experimental results see Supplementary Information and refs 8,24,30,36). Due to the PDA process, a large part of Ti layer was already oxidized before applying electrical stress to play a role of oxygen reservoir. After the electrical forming (Fig. 5a, with positive voltage on the top electrode), more Ti close to Ti/HfO_2_ interface is oxidized to TiO_x_ to generate enough V_O_ (white circles) in the HfO_2_ film to form the filament. However, the V_O_ distribution is unlocalized over the whole planar film structure and the filament might form randomly for different cycles. In Fig. 5b, after the reset process (with a negative voltage on the top electrode), the filament is ruptured so that the HRS state is reached. However, due to the V_O_ distributed in a large area, the V_O_ density is hard to control and the HRS could vary a lot from one cycle to another. Figure 5c,d show the material details of set and reset states of the nanotip device, respectively. No PDA process was carried out therefore the Ti layer stayed mainly un-oxidized before electrical stress (see Fig. 1). After the first cycle of the set process (Fig. 5c, with a negative voltage on the top electrode), mainly the anodic CoSi/HfO_2_ interface is oxidized so that the root of the CF is situated in the present case mostly at the bottom electrode. For Ti, only the part above the nanotip surface is oxidized. The filament is well confined along a straight path way from the nanotip surface towards the planar Ti/TiN top electrode due to the localized electrical field and ionic current density confinement (see Figs 2 and 3). No additional V_O_ is formed in other parts of the Ti/HfO_2_ interface and the HfO_2_ film. Therefore, after reset process as shown in Fig. 5d (with positive voltage on the top electrode and rupture of the narrow constriction on the top electrode), the V_O_ density and distribution can be well controlled by the localized electric field enhancement effects of the nanotip approach so that stable HRS is observed (Fig. 4).

In conclusion, we developed a RRAM device for the embedded memory application by integrating a nanotip as a bottom electrode for the geometric confinement of the V_O_ distribution and nanofilament location. Finite element method simulations evidence a local confinement of the electrical field as well as the ionic current density, which reaches a maximum value at the nanotip edge regions, by using the Si nanotips as the nanoelectrode area. TUNA studies confirm the nanofilament formation above the nanotip electrodes. The nanotip based devices show good RS properties including the forming-free feature, the stable endurance and retention etc. These behaviours are all thanks to the confinement of the V_O_ distribution and the filament location by the localized electrical field distribution and ionic current density. Our results demonstrate a route to CMOS compatible devices and an effective way to control the cycle-to-cycle resistance switching in the RRAM technology. Future works will focus on two topics: 1) to image the space charge distribution in ON- and OFF-states in nanotip devices for a detailed insight into the V_O_ distribution by using TEM electron holography and 2) to setup an array of individually addressable Si nanotip electrodes to integrate this concept into a viable test module for the technological testing.

## Methods

### Fabrication of TiN/Ti/HfO_2_/CoSi_2_/Si tip devices

Devices were processed in a standard 0.25 μm CMOS process line. The Si-tip structure in a square pattern (with tip-tip distance of 1.41 μm) were fabricated using modern advanced lithography on a p^+^-doped (Boron) Si wafer. The fabrication details were explained in ref. 49. The tip diameter can be adjusted by chemical mechanical polishing (CMP) and in this study it is ~120 nm; afterwards the thermal diffusion of Co enabled the selective formation of a CoSi_2_ metal layer (bottom electrode). HfO_2_ (9 nm) was then deposited by atomic vapour deposition at 320 °C, which results in amorphous HfO_2_ films^37^. Finally, a thin Ti (7 nm) scavenging layer and a TiN layer (top electrode) were sputtered on top of HfO_2_. The top electrode size is 1109 μm × 600 μm, which covers an area including ~5200 nanotips (in this case the tip-tip distance is ~11.3 μm).

### TEM measurements

Structural and chemical information are monitored with scanning transmission electron microscopy (STEM) and energy-dispersive x-ray spectroscopy (EDX) using a FEI Tecnai Osiris equipment operated at 200 kV.

### Simulations

Numerical calculations using finite element method (FEM) were performed. Both the electrical field and ionic current density distributions were calculated. A fine mesh consisting of roughly 22000 triangular elements with radius between 0.1 and 1 nm was used in order to ensure convergent results up to small distances from the tip edge.

### Resistance switching

The electrical characterization of the devices was achieved with a Keithley SCS4200 semiconductor analyser using the I-V sweep and the pulse modes. CoSi_2_ was grounded; the voltage was applied on TiN.

### TUNA

In order to image the confined leakage currents, tunnelling atomic force microscopy (TUNA) was carried out using a Pt-Ir coated tip. TUNA works similarly to conductive AFM (C-AFM) but has higher current sensitivity. TUNA is able to characterizes ultra-low currents (<1 pA) through the insulating films. In this case, a device was processed up to the HfO_2_ layer (no top electrode). The TUNA measurements were performed on a thin HfO_2_ (3.4 nm) layer deposited by atomic layer deposition at 300 °C.

### Remark

The TEM and TUNA images have been performed on a device with 1.41 μm tip-tip separation (along Si [100] azimuth) in order to have reasonable AFM statistics and facilitate the TEM sample preparation. The electrical measurements have been performed on a device with 16 μm tips separation.

## Additional Information

**How to cite this article**: Niu, G. *et al*. Geometric conductive filament confinement by nanotips for resistive switching of HfO_2_-RRAM devices with high performance. *Sci. Rep*. **6**, 25757; doi: 10.1038/srep25757 (2016).

## Supplementary Material

Supplementary Information
