# Supplementary material for: Geometric conductive filament confinement by nanotips for resistive switching of HfO2-RRAM devices with high performance
Source: Sci Rep. 2016 May 16;6:25757. doi: 10.1038/srep25757 (PMC4867633; doi:10.1038/srep25757)
Supplement: Supplementary Information [file srep25757-s1.doc]

Supplementary Information

Geometric conductive filament confinement by nanotips for resistive switching of HfO_2_-RRAM devices with high performance

Gang Niu,^1,2*^ Pauline Calka,^1^ Matthias Auf der Maur,^3^ Francesco Santoni,^3^ Subhajit Guha,^1^  Mirko Fraschke,^1^ Philippe Hamoumou,^4^ Brice Gautier,^4^ Eduardo Perez,^1^ Christian Walczyk,^1^ Christian Wenger,^1^ Aldo Di Carlo,^3^ Lambert Alff,^5^ Thomas Schroeder^1,6^

^1^IHP GmbH – Leibniz institute for innovative microelectronics, Im Technologiepark 25, 15236 Frankfurt (Oder), Germany

^2^Electronic Materials Research Laboratory, Key Laboratory of the Ministry of Education & International Center for Dielectric Research, Xi'an Jiaotong University, Xi'an 710049, China.

^3^Dept. Electronics Engineering University of Rome "Tor Vergata" via del Politecnico 1, 00133 Roma, Italy

^4^Institut des Nanotechnologies de Lyon (INL), UMR CNRS 5270, INSA de Lyon, 7 Avenue Jean Capelle, 69621 Villeurbanne, France

^5^Institute of Materials Science, Technische Universität Darmstadt, 64287 Darmstadt, Germany

^6^Brandenburgische Technische Universität, Konrad-Zuse Strasse 1, 03046 Cottbus, Germany

*E-mail: [gangniu@mail.xjtu.edu.cn](mailto:gangniu@mail.xjtu.edu.cn)


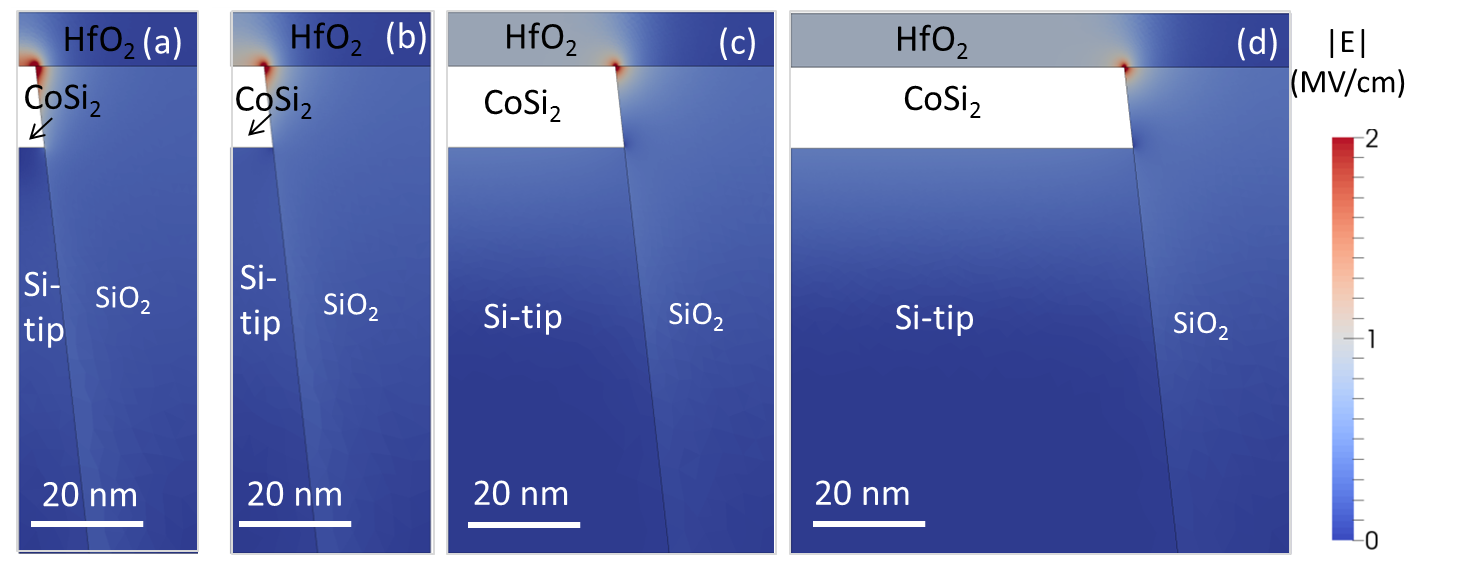


Figure S1. Electrical field (|E|) distribution maps calculated by Finite element method (FEM) for Ti/HfO_2_/CoSi_2_/Si-tip heterostructure with different tip radius of (a) 3 nm; (b) 6 nm; (c) 30 nm and (d) 60 nm. The enhancement of |E| on the Si tip particularly at the edge of the tip can be clearly observed.

Figure S2. Current-Voltage (I-V) characteristics of 1T1R RRAM devices consist of TiN/Ti/HfO_2_/CoSi_2_/planar-Si MIM stacks with a cell size of 500×500 nm^2^. (a) The equivalent circuit depicting the used terminals; BL: bit line, WL: word line, I_D_: drain current, V_S_: source voltage, GND: ground. More details can be found in Ref. 8. (b) 100 cycles I-V curves for Reset process with V_WL_=2 V. (c) 100 cycle I-V curves for Set process with V_WL_=1.4 V. (d) The variation of I_BL_ taken at V_BL_-set=0.2 V as a function of cycles, showing a large OFF state (blue circles) fluctuation, which was strongly improved in nanotip devices with geometric conductive filament confinement.
